# Acute toxicity of metals in *Rhinella diptycha* and *Leptodactylus fuscus* at different temperatures: a perspective for tropical tadpoles

**DOI:** 10.1007/s10646-026-03087-8

**Published:** 2026-06-19

**Authors:** Juliane Silberschmidt Freitas, e Renan Nunes Costa

**Affiliations:** 1 Bauru School of Sciences, São Paulo State University (Unesp), Bauru, São Paulo Brazil; 2 Department of Biological Sciences, Minas Gerais State University, Carangola, Minas Gerais Brazil

**Keywords:** Amphibians, thermal stress, lethal effects, abnormalities, risk assessment

## Abstract

Amphibians are highly vulnerable to environmental pollutants, especially during the larval stage, when temperature fluctuations in ephemeral ponds can interact with metals and alter their toxicity, a scenario that may be intensified by ongoing climate change. This study investigated the toxicity of five metals on tadpoles of *Rhinella diptycha* and *Leptodactylus fuscus*, across two different temperatures (28 and 34 °C). Lethal concentrations for 50% of the population (LC50) were determined to CdCl_2_, CuSO_4_, PbNO_4_, ZnCl_2_ and NiSO_4_, under 28 and 34 °C, for 96 h. Species sensitivity distribution (SSD) curves were then constructed for each metal using LC50 data for both tropical species, together with those from the literature on other tadpole and standard fish species. Toxicity ranking followed Cu > Cd > Zn > Pb > Ni in *L. fuscus* and Cu > Zn > Cd > Ni in *R. diptycha*. Pb LC50 was not calculated in *R. diptycha* due to insufficient number of tadpoles. In almost all cases, increased temperature enhanced metal toxicity in a dose-dependent effect. SSD revealed *R. diptycha* and *L. fuscus* as among the most sensitive species to metals. Body edema was observed in tadpoles exposed to Pb after acute exposure. Our findings show that metal toxicity can be amplified at higher temperatures, reinforcing the importance of accounting for temperature effects when establishing safe environmental concentrations of potential contaminants, particularly for species inhabiting warmer areas.

**Supplementary Information:**

The online version contains supplementary material available at 10.1007/s10646-026-03087-8.

## Introduction

Metals are naturally occurring elements in the environment that can be found in several compartments, including aquatic ecosystems. At low concentrations, metals such as iron (Fe), copper (Cu), and manganese (Mn) play beneficial roles for most organisms, as they are essential for various biochemical and physiological processes. However, several anthropogenic activities, such as agriculture, mining, and industrial processes, can increase metal concentrations in natural systems, making them some of the most widespread contaminants on the planet (Aigberua et al. 2018). Environmental contamination by metals is of particular concern for wildlife due to their persistence in soil and water, as well as their high potential for bioaccumulation, which can disrupt entire food chains (Velusamy et al. 2014; Aigberua et al. 2018).

In addition to human practices that routinely increase metals concentration in the environment, Brazil has experienced impactful mining-related environmental disasters in recent years. Notably, the 2015 Fundão dam collapse in Mariana released approximately 50 million m³ of tailings into the Rio Doce Basin (Girotto et al. 2020), while the 2019 Brumadinho disaster caused 270 fatalities and released ~ 180,000 m³ of tailings. Despite these events, several mining dams remain classified as high risk, and increasingly relaxed environmental regulations continue to threaten biodiversity and human health.

Some organisms are particularly sensitive to metal contamination, and amphibians are especially vulnerable due to their highly permeable skin and their occurrence in small aquatic habitats where pollutants may accumulate (Greulich and Pflugmacher 2003; Yan et al. 2008; Freitas et al. 2022). Furthermore, amphibian metamorphosis and developmental stability can be strongly influenced by environmental factors, including biotic, abiotic, and anthropogenic stressors such as predation, population density, water pH, temperature, and chemical exposure (Denver 1997; Freitas et al. 2016; Costa et al. 2017). Several amphibian species have been documented in anthropized areas and the presence of contaminants in their habitats has been directly linked to population declines worldwide (Hayes et al. 2010; Egea-Serrano et al. 2012; Thambirajah et al. 2019; IUCN 2025). This concern is reinforced by the latest red list published by the International Union for Conservation of Nature (IUCN), which reports that at least 41% of amphibian species are threatened, with highly endemic regions such as Brazil’s Atlantic Forest particularly at risk (Luedtke et al. 2023).

Exposure to metals can disrupt metamorphosis, impair physiological and immune functions, induce malformations, cause oxidative stress and genotoxicity, and negatively affect amphibian behavior and reproduction (Calfee and Little 2017; Do Amaral et al. 2019; Pinelli et al. 2019; Carvalho and Pinto-Vidal 2024; Costa et al. 2024). Despite evidence of their multi-pathway toxicity to amphibians, critical baseline data for metals, such as environmentally relevant concentration thresholds for most species, remain unknown. Additionally, the lack of standardized protocols for ecotoxicological assays on tropical amphibian larvae has led to the use of international guidelines developed for species from temperate regions, as Amphibian Metamorphosis Assay (AMA) (OECD, 2009) and Frog Embryo Teratogenesis Assay (FETAX) (NTP 2000), resulting in experimental conditions that often do not reflect the species’ natural habitats. Similarly, ABNT NBR 15,088 and ABNT NBR 15,499, developed for fish and commonly used as surrogate protocols to assess chemical lethality in tadpoles, prescribe an environmental temperature of 25–26 ± 2 °C. Nonetheless, temperatures of up to 42 °C have been recorded in habitats of native tadpoles in Brazil (Freitas et al. 2016; unpublished data collected between 2024 and 2025), and similar conditions are likely to occur in other warm regions worldwide, particularly in temporary ponds that serve as breeding sites for many amphibian species.

Water temperature can critically influence chemical toxicity in tadpoles and other aquatic animals (Hallman and Brooks 2016; Freitas et al. 2016, 2017, 2022). In ectotherms, higher temperatures accelerate metabolism, often increasing toxicity within specific thermal ranges (Leung et al. 2000; McConnachie and Alexander 2004; Kwok and Leung 2005), though the magnitude of this effect varies among taxa due to physiological and thermal differences. Temperature fluctuations, particularly at thermal extremes, can modify the toxic effects of chemicals, with cascading impacts from individuals to ecosystems (Cairns et al. 1975, 1978; Lau et al. 2014; Zhou et al. 2014). Thus, understanding these interactions in amphibians is especially important in the context of climate change scenarios, as well emphasized by the Intergovernmental Panel on Climate Change (IPCC) in recent years. Despite their ecological importance, the combined action of metals and temperature on amphibians remain poorly understood, hindering survival predictions and the development of effective Environmental Risk Assessments (ERAs) and conservation strategies for this vulnerable group.

This study evaluated lethal toxicity of five metals, cadmium (Cd), copper (Cu), zinc (Zn), nickel (Ni), and lead (Pb), in two Brazilian tadpoles, *Rhinella diptycha* (Bufonidae) and *Leptodactylus fuscus* (Leptodactylidae), under two different temperatures (28 °C and 34 °C). To date, lethal and hazard concentrations for these metals remain unknown for both species, as well as for most tropical tadpoles, particularly under environmentally relevant conditions. It was hypothesized that elevated temperatures would increase metal toxicity in both species, reflecting their species-specific metabolic responses and thermal tolerances. A species sensitivity distribution (SSD) curve was also constructed using data from this study combined with literature data from other tadpole species, to compare sensitivity to metals and to estimate hazard concentrations for amphibians.

## Materials and methods

### Tadpoles’ species

Spawns of *Rhinella diptycha* and *Leptodactylus fuscus* (three and five, respectively) were collected from different water bodies during the rainy season in the Ituiutaba region, Minas Gerais, Brazil. These sites are located in a non-agricultural matrix, considered a preserved area in the region, with no evident human activity. After hatching, larvae were acclimatized and maintained under laboratory conditions (27 ± 2 °C, pH 7.5 ± 1 and constant aeration), where they were fed daily with food for tropical fish until they reached Gosner stage 25 (Gosner 1960). Tadpoles had their water partially renewed twice a week and were kept under a 12:12 h light–dark photoperiod. Both species were collected under license n. 85552-1 provided by the Biodiversity Authorization and Information System (SISBio). Both amphibian species are widely distributed in Brazilian Cerrado and are not currently at risk of extinction or experiencing population declines, being considered representative tropical species in Brazil as they exhibit wide geographic distribution across the country and are commonly in anthropized areas (AmphibiaWeb 2025; IUCN 2025).

### Acute toxicity tests

Acute toxicity tests were conducted at the Center of Study and Research in Aquatic Ecotoxicology, CEPEA, located in Ituiutaba, Minas Gerais, Brazil. All toxicity tests were carried out using the temperatures of 28 and 34 °C, corresponding to the thermal gradients recorded by data loggers placed into water bodies during the summer, which is the peak reproductive season for amphibians in Brazil (Freitas et al. 2016, 2017a, b, 2022). Water parameters and photoperiod during the acute toxicity tests were the same as those used during the acclimation period (i.e. 27 ± 2 °C, pH 7.5 ± 1, 12:12 h light–dark photoperiod).

Static acute exposures were conducted over a period of 96 h with no tadpole feeding. Five nominal concentrations of the metals CdCl_2_, CuSO_4_, Pb(NO_3_)_2_, ZnCl_2,_ and NiSO_4_ (Sigma-Aldrich, ≥ 98- 99.99% purity) were tested in each species at temperatures of 28 and 34 °C. Experiments with Pb(NO_3_)_2_ were not conducted with *R. diptycha* due to an insufficient number of individuals required for the assay. For each temperature, a control group (with no contaminant) was used to assess the isolated effect of temperature on the survival of the animals. Each metal concentration was represented by three replicates containing five tadpoles each one (*n* = 15 tadpoles/concentration). Sterile cylindrical plastic pots of 1 L capacity containing 750 mL of solution were used as replicates. Based on concentration data for other tadpole species from the U.S. Environmental Protection Agency (US-EPA) database, baseline values were established for LC50 calculations. The concentrations used for *L. fuscus* were: 1, 1.45, 1.8, 2.55, and 2.7 mg/L for CdCl_2_; 0.025, 0.05, 0.1, 0.2, and 0.4 mg/L for CuSO_4_; 0.5, 1.0, 2.0, 3.0, and 5.0 mg/L for NiSO_4_; 0.625, 1.25, 2.5, 5.0, and 10.0 mg/L for Pb(NO_3_)_2_ and 0.25, 0.5, 1.0, 2.0, and 3.0 mg/L for ZnCl_2_. Concentrations for *R. diptycha* were 2.40, 2.55, 2.70, 2.85 and 3.0 mg/L for CdCl_2_; 0.025, 0.05, 0.10, 0.20, and 0.4 mg/L for CuSO_4;_ 1.5, 2.25, 4.0, 8.0, 15.0 mg/L for NiSO_4,_ and 0.25, 0.5, 1.0, 2.0, and 3.0 mg/L for ZnCl_2_.

To ensure that all replicates reached the same temperature simultaneously, manually regulated thermostats were used to control thermal gradients in a system of water baths. In this setup, the 1-liter replicates were placed inside a larger water container (20 L) that was partially filled. Temperatures were gradually adjusted to prevent thermal shock stress in the animals and then monitored twice a day throughout the 96-hour experiment. Mortality was verified daily and the physicochemical characteristics of the water (pH, conductivity, and dissolved oxygen) were recorded at the beginning and at the end of the test. Surviving tadpoles were anesthetized and euthanized by immersion in a benzocaine solution (100 mg/L; Sigma-Aldrich, purity > 99%) and subsequently preserved in 10% formalin for assessment of external body morphology. The lethal concentration of 50% population (CL50) during 96 h was calculated using Probit Analysis.

### Metals quantification

Concentrations of metals in the solutions prepared for toxicity tests were assessed using water samples prepared at the beginning of the experiments. Chemical quantifications were conducted at the Department of Hydraulics and Sanitation (SHS) of the São Carlos Engineering School (ESSC), according to the 24th edition of Standard Methods. Detection of Cd, Cu, Ni, Zn, and Pb followed Standard Methods for the Examination of Water and Wastewater (SMWW) 3111B by direct atomic absorption spectrophotometry, AA 240FS VARIAN, in which each metal absorbs a specific wavelength. The energy absorbed by the sample at a particular wavelength increased proportionally with the concentration of the element present (Miranda 2017). Table S1 presents the metals quantified in the samples and the detection limits for each element.

### Species sensitivity distribution (SSD)

To evaluate and compare the sensitivity of tadpole species to the studied metals, Species Sensitivity Distribution (SSD) curves were constructed using LC50_96h_ values obtained from the acute toxicity tests, along with corresponding data for other amphibian species available in US-EPA ECOTOX database (US EPA, 2025) (https://cfpub.epa.gov/ecotox/*).* Data were then verified through searches in SciELO, Google Scholar, and PubMed to confirm information. A LC50_96h_ average was calculated when multiple studies were available for the same species and metal. The acute toxicity values extracted from the aforementioned database were standardized and grouped according to duration of exposure (96 h exposure), developmental stage (larvae or tadpole), and type of exposure (exposure in water). Given that metals can occur in various formulations or salt forms, data corresponding only to products with the same CAS Registry Number as the chemicals used in this study were selected.

ETX software version 3.0 was used to construct the log-normal distribution of the values and to calculate the 5th and 50th percentile with their confidence limits (Aldenberg and Jaworska 2000). Log-normality was tested by the Anderson-Darling test, which was evaluated at 5% significance level. Safety limits with their lower and upper limit of confidence were also calculated to determine concentrations that could protect 95% of the tadpole species (hazard concentration for 5% of population – HC_5_) (Maltby et al. 2005; Kwok et al. 2007). To identify Brazilian native species among the toxicity data available through the search system, the online system Amphibian Species of the World (Frost 2025) was used. LC50_96h_ ​​found for *R. dypticha* and *L. fuscus* at both temperatures were included in the SSDs and highlighted in the curves to illustrate how species sensitivity may vary when temperature is taken into account.

In the second step of our sensitivity analysis, the current perspective on ERAs for aquatic vertebrates was considered, which suggests that amphibians would be inherently safeguarded during their larval stages by the protection of sensitive fish species. To this end, acute toxicity (LC50_96h_) from US-EPA database for the standard species of fish, *Danio rerio*,* Pimephales promelas*,* Oryzias latipes*, and *Oncorhynchus mykiss* were included into the SSD analysis. Here, only the 96-h LC50 values at 28 °C for *R. dypticha* and *L. fuscus* were considered, in order to better standardize the comparison of metal sensitivity by excluding thermal stress conditions. Data for fish were grouped according to duration of exposure (96 h exposure) and developmental stage (larvae, juvenile and adult fish). Results with eggs were excluded from the toxicity data for both amphibians and fish.

## RESULTS

### Acute toxicity

**Fig. 1:**
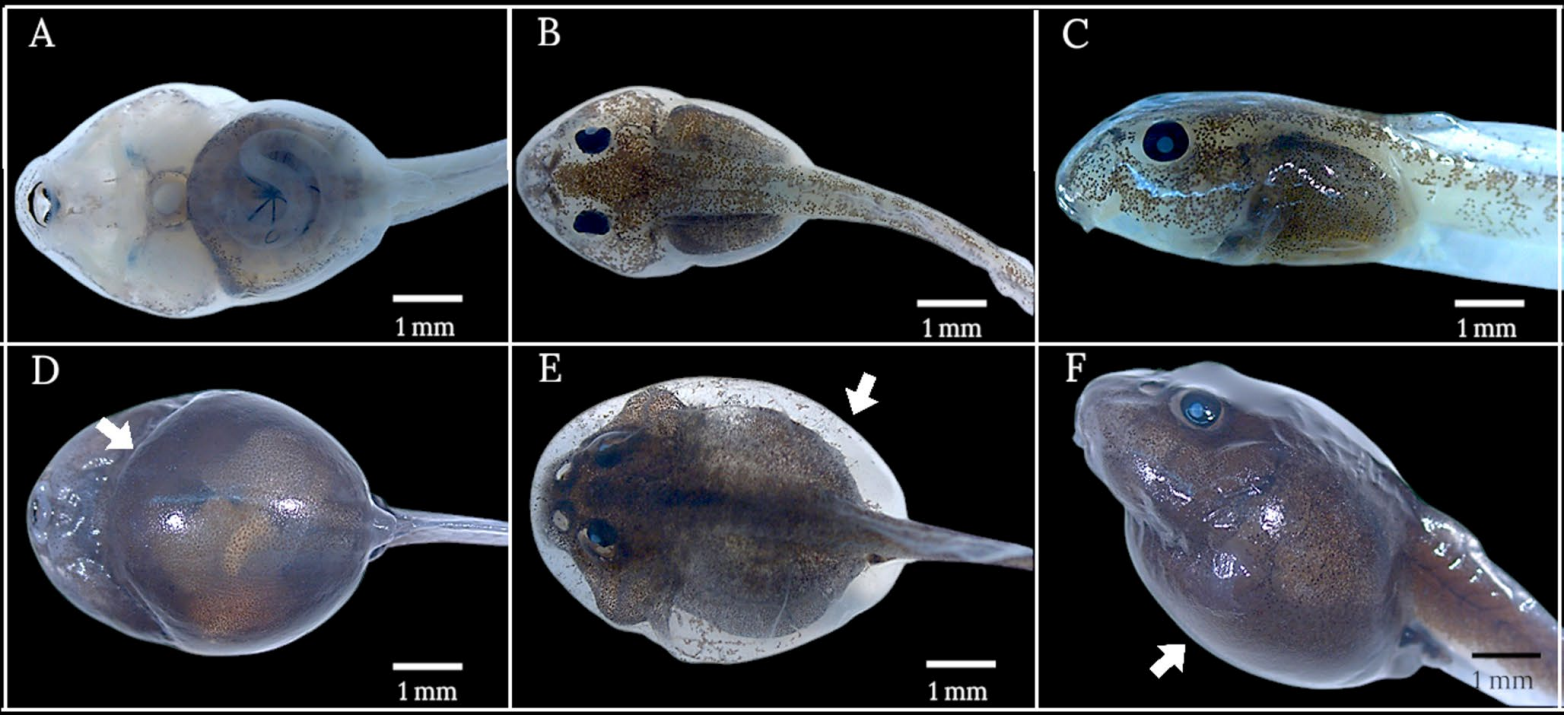
Figures A, B and C show tadpoles from the control group, with no evidence of abnormalities, and figures D, E and F indicate edema by the black arrow

No mortality was observed in the control groups of either tadpole species at 28–34 °C throughout the 96-hour experimental period, indicating that thermal stress alone did not induce lethality under the tested conditions. After exposure to metals, treatments caused dose-dependent mortality after 96 h for both species. Numerous Pb-exposed tadpoles exhibited edema (Fig. 1). A temperature-dependent toxicity was also observed for almost all metals. In *L. fuscus*, metals followed the toxicological relationship Cu > Cd > Zn > Pb > Ni (more to less toxic) for both tested temperatures. LC50_96h_ in this species were 0.086, 1.19, 2.05, 2.84 and 4.81 mg/L for Cu, Cd, Zn, Pb and Ni, respectively at 28 °C. At 34 °C, these values were 0.092, 0.20, 1.67, 1.86 and 2.74 mg/L, respectively (Table 1). With emphasis on the metals Cd and Ni, a notable increase in toxicity of 5.95 times and 1.7 times, respectively, was observed in animals maintained at the highest temperature.

In *R. diptycha*, the toxicity of metals followed the order from most to least toxic: Cu > Zn > Cd > Ni at both temperatures. LC50_96h_ in this species were 0.09, 1.83, 2.83, and 3.08 mg/L for Cu, Cd, Zn, and Ni, respectively at 28 °C. At 34 °C, these values were 0.042, 1.58, 2.21, and 1.93 mg/L, respectively (Table 1). The most pronounced temperature-induced increase in mortality for *R. diptycha* tadpoles was observed with Cu and Ni, with toxicity rising by 2.15-fold and 1.6-fold, respectively. As observed, Cu was the most toxic metal, while Ni exhibited the lowest toxicity for both species. Table S1 and Table 1 show metal concentration quantified in water samples and the LC50_96h_ values ​​of the studied metals at temperatures of 28 and 34 °C, respectively. Most metals exhibited time-dependent toxicity, with LC₅₀ values decreasing over the 24–96 h exposure period, particularly at higher temperatures (Table S2).

**Table 1:** LC50_96h_ of metals CuSO_4_, CdCl_2_, ZnCl_2_, Pb(NO_3_)_2_ and NiSO_4_ in tadpoles at 28 and 34°C. LL: Lower limit; UL: Upper limit.

Metals	*Leptodactylus fuscus*		*Rhinella diptycha*	
	LC50_96h_ (LL - UL) mg/L		LC50_96h_ (LL - UL) mg/L	
	28 °C	34 °C	28 °C	34 °C
CuSO_4_	0.086 (0.05–0.13)	0.092 (0.054–0.15)	0.09 (0.06–0.11)	0.042 (0.03–0.05)
CdCl_2_	1.19 (0.90–1.55)	0.20 (0.007–0.55)	2.83 (2.66–3.00)	2.21 (2.03–2.41)
ZnCl_2_	2.05 (1.29–3.24)	1.67 (1.14–2.46)	1.83 (1.21–2.76)	1.58 (1.01–2.47)
Pb(NO_3_)_2_	2.84 (1.63–4.95)	1.86 (0.98–3.53)	NR*	NR*
NiSO_4_	4.81 (3.13–7.30)	2.74 (2.02–3.72)	3.08 (2.25–4.23)	1.93 (1.35–2.75)

*NR: Non reported

### Species sensitivity distribution (SSD)

First, the search in the US-EPA database did not return any lethal toxicity data for the metals Cd, Cu, Zn, Pb, and Ni for *L. fuscus* and *R. diptycha*. Additionally, no toxicity data were reported for any Brazilian species, with the exception of Zn, for which data were available for *Rhinella arenarum*. Considering the number of toxicity records across global tadpole diversity, acute lethal effects have been analyzed in 21 species for CuSO₄, 10 species for CdCl₂, 5 species for Pb(NO₃)₂, and only one species for NiSO₄ and ZnCl₂.

**Fig. 2:**
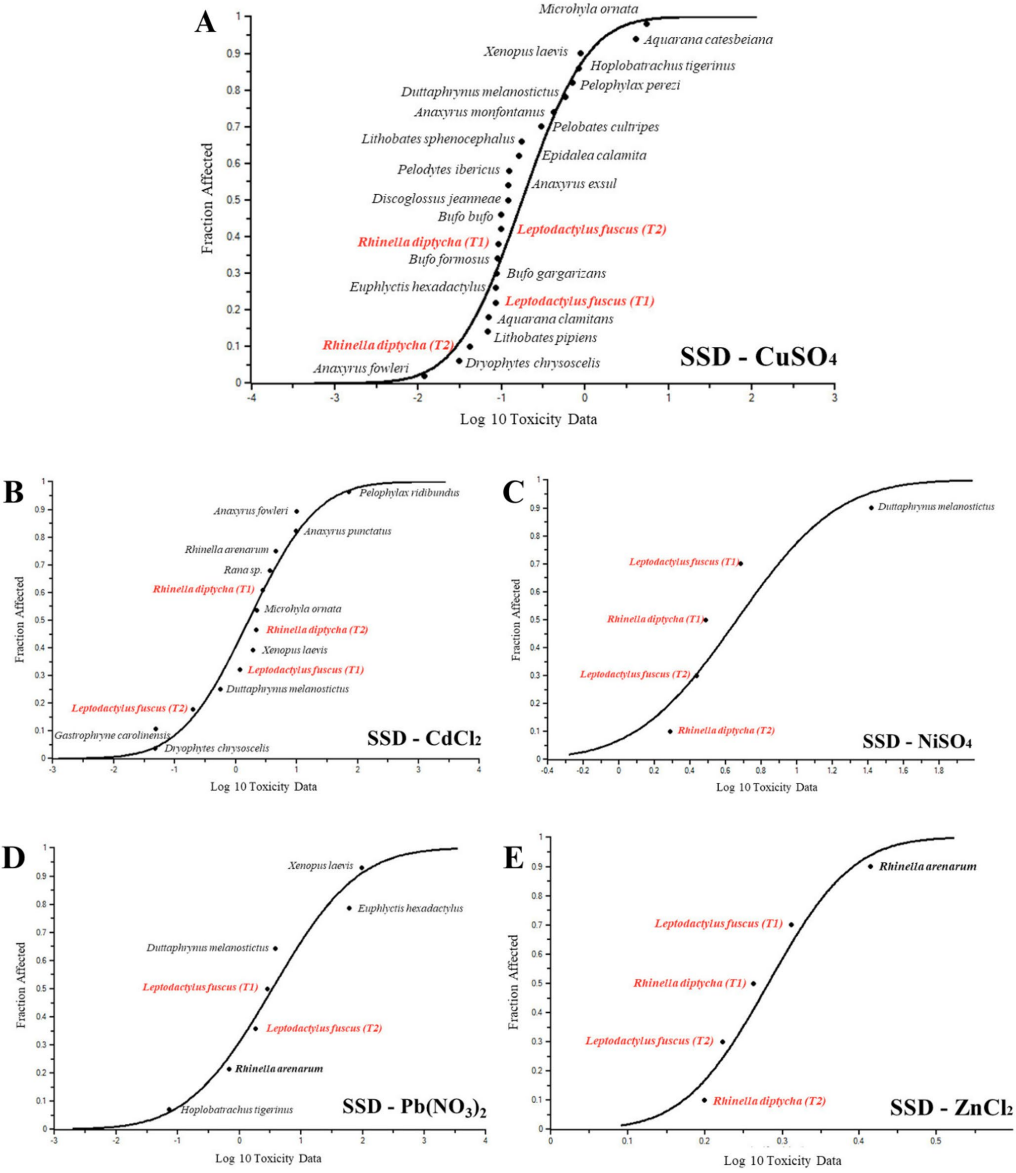
Species sensitivity distribution (SSD) for CuSO_4_ (A), CdCl_2_ (B), NiSO_4_ (C), Pb(NO_3_)_2_ (D) and ZnCl_2_ (E) in amphibians, considering only larval aquatic stages. Data of 96 h-LC50 found in this study for *Rhinella dypticha* (red) and *Leptodactyllus fuscus* (blue) were included into the SSD considering the temperatures of 28 (T1) and 34 °C (T2). Captions in bold correspond to Brazilian species

Considering the SSDs for amphibians during their larval stages, *R. diptycha* and *L. fuscus* were either the most sensitive species for Zn and Ni or among the three most sensitive species considering the other metals. When temperature variation was taken into account, the positions of *R. diptycha* and *L. fuscus* also shifted in the SSDs (Fig. 2), with the highest temperature increasing their sensitivity. Hazard Concentration for 5% of amphibian population (HC5) for each metal, as well as their lower and upper limit of the HC5 (95% confidence intervals) are compiled in Table 2.

**Table 2:** Hazard concentration for 5% of amphibian population (HC_5_) at larval stages (left column) and amphibians and fish population (right column) to CdCl_2_, CuSO_4_, NiSO_4_, Pn(NO_3_)_2_, and ZnCl_2_ and their respective lower (LL) and upper (UL) confidence limits (95% confidence intervals). Only larval stages were considered

Metal	Tadpoles HC_5_ (mg/L) (LL - UL)	Tadpoles and Fish HC_5_ (mg/L) (LL - UL)
CdCl_2_	0.0539 (0.008–0.176)	0.067 (0.014–0.193)
CuSO_4_	0.0166 (0.006–0.031)	0.0141 (0.005–0.028)
NiSO_4_	0.739 (0.061–1.98)	1.07 (0.230–2.398)
Pb(NO_3_)_2_	0.046 (0.0007–0.347)	0.036 (0.001–0.240)
ZnCl_2_	1.347 (0.835–1.629)	1.078 (0.230–2.398)

**Fig. 3:**
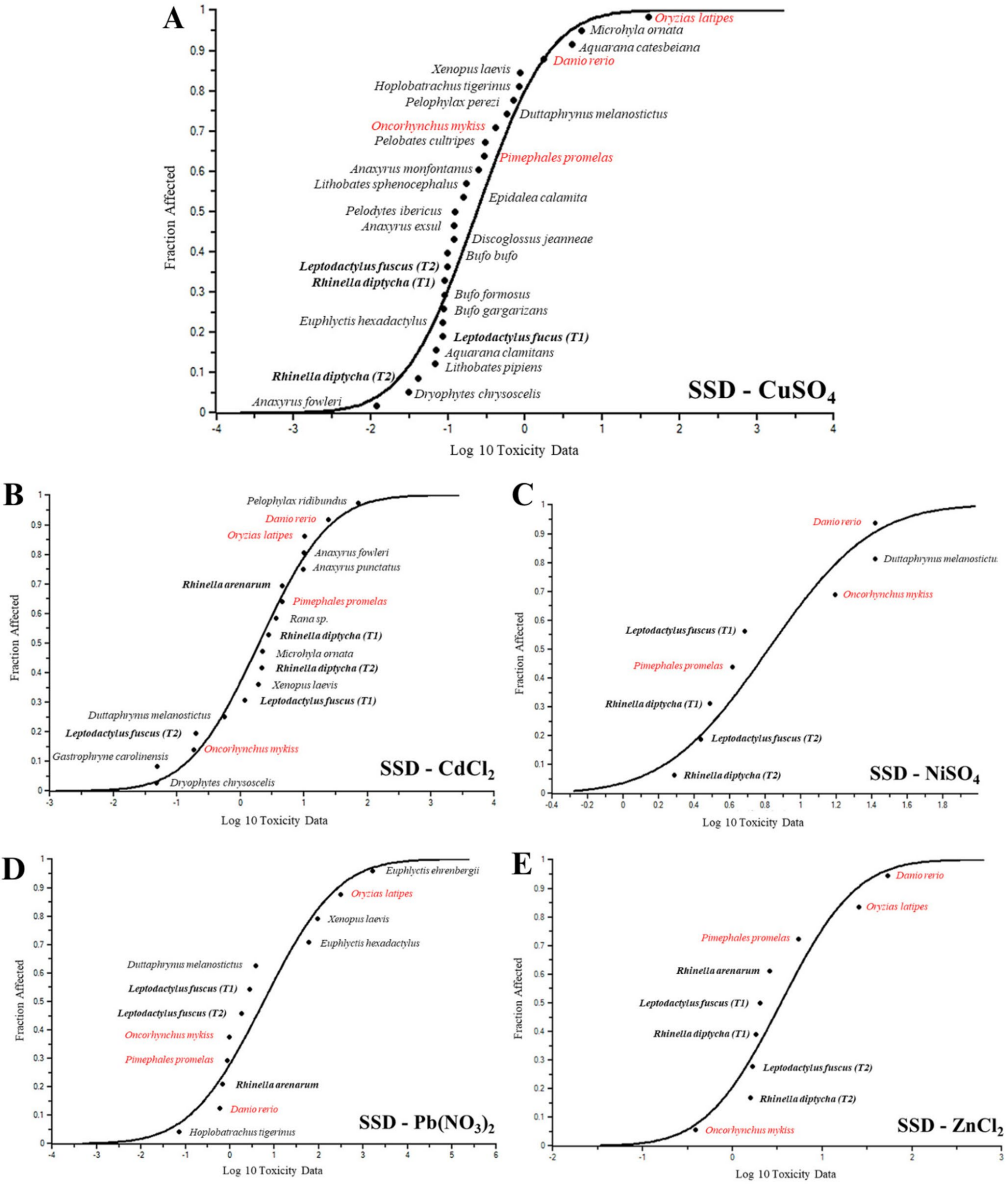
Species sensitivity distribution (SSD) for CuSO_4_ (A), CdCl_2_ (B), NiSO_4_ (C), Pb(NO_3_)_2_ (D) and ZnCl_2_ (E), considering acute toxicity data for tadpoles and fish of model species used in toxicological assays. Captions in bold correspond to native Brazilian species, and those in red to fish species

The SSDs constructed using LC50_96h_ metal toxicity data for fish and tadpoles, excluding thermal stress effects, revealed significant interspecific variation in their sensitivities. Amphibians during their larval stages were more sensitive to CdCl_2_, CuSO_4_, and Pb(NO_3_)_2_, while some fish species were more sensitive to NiSO_4_ and ZnCl_2_ (Fig. 3). For CuSO_4_, tadpole species exhibited greater sensitivity compared to all fish species. Substantial variation in the sensitivity of model fish species to different metals was also evident. HC5 of amphibians and fish population for each metal, as well as their lower and upper limit of the HC5 (95% confidence intervals) are compiled in Table 2.

## Discussion

Our results indicated that higher temperatures may increase metal toxicity in tadpoles, as lethality at 34 °C was greater than at 28 °C for most elements in both species, *L. fuscus* and *R. diptycha*. These findings align with previous studies on aquatic organisms, which reported increased metal toxicity at elevated temperatures in 71% of microalgae, 86% of zooplankton, and 77% of fish (Nin and Rodgher 2021). Thus, based on IPCC projections for the end of the 21st century, climate warming could potentially amplify the effects of metal contamination across multiple trophic levels. Despite the growing attention to temperature–chemical interactions in the ecotoxicology area in recent years, experimental data on how rising temperatures may affect amphibian mortality remain extremely limited or altogether absent. This is particularly concerning because (1) standard experimental protocols available for amphibians can be underestimating the toxicity of various contaminants to species from tropical regions, where baseline temperatures are naturally higher; and (2) it highlights concerns about the current global scenario, characterized by rising average temperatures alongside escalating environmental contamination.

The toxicokinetics of metals in water are strongly influenced by physicochemical factors of the environment, such as pH and water hardness (Carvalho and Pinto-Vidal 2024). pH influences metal solubility in water by altering the balance of H⁺ and OH⁻ ions, thereby affecting chemical dynamics. Low pH generally increases metal solubility and bioavailability, enhancing toxicity (De Paiva Magalhães et al. 2015; Väänänen et al. 2018), while carbonate (CO_3_^−2^) and bicarbonate (HCO_3_) in alkaline waters can precipitate metals, reducing their bioavailability (Davies et al. 1993; De Paiva Magalhães et al. 2015). Increased water hardness often decreases metal toxicity due to competition between Ca²⁺ and Mg²⁺ at binding sites, rather than through changes in metal bioavailability (Pascoe et al. 1986; Diggs and Parker 2009; Ebrahimpour et al. 2010). In *Rhinella arenarum*, for example, elevated Ca²⁺ concentration in hard water exerted a protective effect on tadpoles exposed to Cd after 24 and 96 h (Mastrángelo et al. 2011). Although the relationship with temperature is less well understood, it is known that warming can alter the partitioning and diffusion of chemicals in the environment, thereby affecting their aggregation, precipitation, speciation, solubility, and bioavailability to organisms (Heugens et al. 2001; Bourgeault et al. 2013).

Higher temperatures also commonly reduce dissolved oxygen (DO) levels in water and, when combined with increased metabolic rates in ectotherms, it may trigger hyperventilation, enhancing metal uptake and favoring bioaccumulation (Lau et al. 2014; Carvalho and Pinto-Vidal 2024). Studies approaching oxygen-limited thermal tolerance (OLTT) models suggested that aquatic ectotherms typically live within a specific temperature range that allows them to perform aerobic metabolism without experiencing significant stress. When temperatures deviate from their optimal range, animals may experience a mismatch between energy demand and supply, shifting metabolism toward anaerobic pathways to maintain essential cellular and physiological functions (Pörtner 2010). Such changes can alter total and basal metabolic rates, increasing susceptibility to chemical stressors. Elevated temperatures may also affect gene expression, disrupt hormonal and enzymatic activity, and induce oxidative stress (Leung et al. 2000; Morley et al. 2012). Antioxidant responses in tadpoles are typically activated under warming conditions; however, if these protective mechanisms are suppressed, individuals may experience significant physiological impairment, potentially leading to irreversible damage (Freitas and Almeida 2016).

Accelerated metabolism as a consequence of raised temperatures may augment reactive oxygen species (ROS) production (Lushchak 2011), which at high levels can cause oxidative injury to lipids, proteins, carbohydrates, and even DNA (Toyokuni 1999; Abele and Puntarulo 2004; Halliwell and Gutteridge 2007). In the tropical tadpole species *Physalaemus nattereri*, increased temperature from 28 to 36 °C impaired antioxidant enzymes catalase (CAT), glutathione-S-transferase (GST), glutathione reductase (GR) and glucose-6-phosphate dehydrogenase (G6PDH), compromising their antioxidant defense system (Freitas and Almeida 2016). Previous studies on *P. nattereri* and *R. diptycha* tadpoles showed pronounced activation of their antioxidant systems when heat stress was combined with pesticide exposure (Freitas et al. 2017a, b). The synergistic effect of temperature with environmental contaminants on antioxidant responses was also observed in the bullfrog tadpole *Aquarana catesbeiana* exposed to 2-hydroxyatrazine, a primary degradation product of the herbicide atrazine (Carneiro et al. 2023). Undoubtedly, tropical tadpoles have biochemical and physiological mechanisms that ensure their survival in warmer waters. However, the presence of a chemical stressor in the environment is likely to overwhelm the tadpoles’ defense mechanisms, increasing the risk of mortality, as metals may also interfere with biological molecules and redox reactions (Ossana et al. 2013; Pérez-Iglesias et al. 2019; Costa et al. 2024). This may help clarify some of the mechanisms underlying the increased metal toxicity observed in tadpoles exposed to different elements at 34 °C compared with 28 °C in our study.

Tadpoles can bioaccumulate metals by direct absorption across their gills and skin, and by ingesting contaminated sediment during foraging (Yologlu and Ozmen 2015; Wei et al. 2015; Dubaissi et al. 2018; Guezgouz et al. 2021). Their gills are particularly effective at metal uptake because the epithelial surface is negatively charged by phospholipids, facilitating attraction of metal ions from the surrounding water (Wittmann 1981; Pagenkopf 1983). Once inside the organism, metals enter cells through ionic and molecular mimicry by using transporters meant for essential ions and organic molecules, as well as by some carrier proteins metals (Ballatori 2002; Bridges and Zalups 2005). As mentioned before, metals can cause a cascade of adverse effects in amphibians, including oxidative stress (Carvalho et al. 2020; Fernandes et al. 2021), increased metallothioneins (Yologlu and Ozmen 2015; Carvalho et al. 2017; Carlsson and Tydén 2018), neurotoxicity (Nunes 2011; Ossana et al. 2013), genotoxicity and mutagenicity (Monteiro et al. 2018; Patar et al. 2021; Costa et al. 2024), disrupted metamorphosis (Sun et al. 2017; Ya et al. 2021; Costa et al. 2024), hepatotoxicity and metabolic alterations (Shi et al. 2018; Ju et al. 2020; Pinto-Vidal et al. 2022), thyroid toxicity (Sun et al. 2017), and behavioral changes (Ranatunge et al. 2012; Hu et al. 2019). Even following acute exposure in our study, numerous Pb-exposed tadpoles exhibited edema (Fig. 1), indicating an excessive liquid accumulation caused by a disruption of osmotic regulation or inflammation in contaminated animals. This external morphological abnormality has already been observed in some metal-exposed tadpoles species, such as *Rana luteiventris* exposed to Cd (Lefcort et al. 1998), *Bufo gargarizans* exposed to Cu (Xia et al. 2012) and *Xenopus laevis* exposed to Zn (Martini et al. 2012).

Despite well-documented metal threats to amphibians, protective concentration thresholds remain poorly defined. Available data in literature are extremely limited, with *R. arenarum* being the only tropical species for which acute Cd and Pb toxicity has been described. Moreover, metal LC₅₀ values for tadpoles across different temperatures are virtually nonexistent. Regarding species sensitivity, SSDs combining fish and amphibian results showed high variation: tadpoles were more sensitive to Cd, Cu, and Pb, whereas fish were more sensitive to Ni and Zn. Some studies have indicated *Oncorhynchus mykiss* as a sensitive fish species and potential surrogate for tadpoles in aquatic ecotoxicology (Daam et al. 2019). However, while *O. mykiss* showed high sensitivity to Cd and Zn, this pattern was not consistent for Ni and Cu. In contrast, *Danio rerio*, the standard aquatic vertebrate in Brazilian regulations (ABNT NBR 15088), was among the more metal‑tolerant species, except for Pb. Currently, European Food Safety Authority (EFSA PPR Panel, 2013) recommends using an assessment factor of 100 with acute LC₅₀ values from *O. mykiss* to protect larval amphibians. For the species and metals investigated in this study, this assessment factor appears adequate to ensure protection under the tested conditions. Nonetheless, from an environmental protection perspective, a more effective alternative to assessing metal risk in aquatic vertebrates would be to integrate HC₅ values from both amphibians and fish species, rather than relying exclusively on fish data.

Given the substantial toxic variability observed across different thermal gradients for the studied metals, it is strongly recommended to include temperature‑based extrapolation factors into environmental risk assessment frameworks. Although an assessment factor of 10 has been proposed as adequate for protecting freshwater ectotherms under tropical conditions (Lau et al. 2014), and this appears consistent with our toxicological results for both tadpole species with respect to the studied metals, its applicability to amphibians remains uncertain due to the scarcity of toxicity data for most species. Elevated toxicity at higher temperatures likely reflects reduced physiological tolerance near thermal limits compared to optimal thermal ranges, and the resulting lower survival of tadpoles may have important implications for population dynamics under climate change (McConnachie and Alexander 2004; Kwok and Leung 2005; Bao et al. 2008; Li et al. 2015; Hallman and Brooks 2016). While this study focused on short-term lethal effects, the results emphasize the need to investigate long-term sublethal impacts of metals under thermal stress. In light of current global warming and the vulnerability of amphibians, these findings advance ecotoxicological knowledge and highlight the importance of expanding experimental studies using representative species and more realistic conditions, including mesocosm‑based approaches. Knowledge of the influence of warming on chemical toxicity and species vulnerability has become increasingly important to address within ecological risk frameworks and conservation strategies, as well to extrapolating current knowledge and its potential impacts on endangered species.

## Supplementary Information

Below is the link to the electronic supplementary material.

Supplementary Material 1

## Data Availability

No datasets were generated or analysed during the current study.
