# Supplementary material for: Acute toxicity of metals in Rhinella diptycha and Leptodactylus fuscus at different temperatures: a perspective for tropical tadpoles
Source: Ecotoxicology. 2026 Jun 19;35(5):124. doi: 10.1007/s10646-026-03087-8 (PMC13282347; doi:10.1007/s10646-026-03087-8)
Supplement: Supplementary file 1 — Supplementary Material 1 [file 10646_2026_3087_MOESM1_ESM.docx]

SUPPLEMENTARY MATERIAL

**Table S1.** Metal concentrations in samples prepared for acute toxicity tests. ND: not detected, indicating concentrations below the detection limit.

| **Metal** | **Selected concentration**  **(mg/)** | **Measured concentration in samples (mg/L)** | **Detection limit**  **(mg/L)** |
| --- | --- | --- | --- |
| CuSO_4_ | 0.025 | ND |  |
|  | 0.05 | ND |  |
|  | 0.1 | 0.049 | 0.018 |
|  | 0.2 | 0.175 |  |
|  | 0.4 | 0.460 |  |
| Pb(NO_3_)_2_ | 0.625 | 0.68 |  |
|  | 1.25 | 1.41 |  |
|  | 2.5 | 2.9 | 0.08 |
|  | 5.0 | 5.42 |  |
|  | 10.0 | 11.35 |  |
| CdCl_2_ | 1 | 0.97 |  |
|  | 1.45 | 1.41 |  |
|  | 1.8 | 1.84 |  |
|  | 2.4 | 2.13 |  |
|  | 2.55 | 2.21 | 0.007 |
|  | 2.7 | 2.71 |  |
|  | 2.85 | 2.83 |  |
|  | 3.0 | 3.11 |  |
| ZnCl_2_ | 0.25 | 0.29 |  |
|  | 0.5 | 0.52 |  |
|  | 1.0 | 1.21 | 0.006 |
|  | 2.0 | 2.3 |  |
|  | 3.0 | 3.31 |  |
| NiSO_4_ | 0.5 | 0.282 |  |
|  | 1.0 | 0.603 |  |
|  | 1.5 | 0.630 |  |
|  | 2.0 | 1.305 |  |
|  | 2.25 | 2.09 |  |
|  | 3.0 | 2.49 | 0.016 |
|  | 4.0 | 3.89 |  |
|  | 5.0 | 4.85 |  |
|  | 8.0 | 7.75 |  |
|  | 15.0 | 15.62 |  |

**Table S2:** LC₅₀ values in *Leptodactylus fuscus* and *Rhinella diptycha* tadpoles exposed to metals at 28 and 34 °C, after 24 and 96h of exposure. LL: lower limits; UL: upper limits. NM: no significant mortality.

| **Metals**  (mg/L) | ***L. fuscus*** | | | |
| --- | --- | --- | --- | --- |
|  | **28°C** | | **34°C** | |
|  | **LC50_24h_ (LL-UL)** | **LC50_96h_**  **(LL-UL)** | **LC50_24h_**  **(LL-UL)** | **LC50_96h_**  **(LL-UL)** |
| CdCl₂ | 2.62  (1.07 - 6.44) | 1.18  (0.90 - 1.55) | 1.16  (0.94 - 1.43) | 0.2  (0.007 - 0.55) |
| CuSO₄ | 0.086  (0.05 - 0.13) | 0.086  (0.05 - 0.13) | 0.092  (0.05 - 0.15) | 0.092  (0.05 - 0.15) |
| ZnCl₂ | 2.76  (1.72 - 4.42) | 2.05  (1.29 - 3.24) | 2.07  (1.34 - 3.19) | 1.67  (1.14 - 2.46) |
| NiSO₄ | NM | 4.81  (3.13 - 7.30) | 5.21  (3.21 - 8.48) | 2.74  (2.02 - 3.72) |
| Pb(NO₃)₂ | 16.22  (15.89 - 17.19) | 2.84  (1.63 - 4.95) | 8.58  (7.89 - 9.12) | 1.85  (0.98 - 3.53) |
|  | ***R. diptycha*** | | | |
| **Metals**  (mg/L) | **28°C** | | **34°C** | |
|  | **LC50_24h_**  **(LL-UL)** | **LC50_96h_**  **(LL-UL)** | **LC50_24h_**  **(LL-UL)** | **LC50_96h_**  **(LL-UL)** |
| CdCl₂ | 3.03  (2.85 - 3.23) | 2.83  (2.66 - 3.00) | 2.50  (2.31 - 2.71) | 2.21  (2.03 - 2.41) |
| CuSO₄ | 0.163  (0.11 - 0.23) | 0.09  (0.06 - 0.11) | 0.225  (0.14 - 0.34) | 0.042  (0.03 - 0.05) |
| ZnCl₂ | 3.18  (1.77 - 5.69) | 1.83  (1.21 - 2.76) | 1.79  (1.13 - 2.84) | 1.58  (1.01 - 2.47) |
| NiSO₄ | 7.44  (5.15 - 10.74) | 3.08  (2.25 - 4.23) | 5.53  (3.93 - 7.73) | 1.93  (1.35 - 2.75) |
